# Obstetric Medicine

**Published:** 1858-11

**Authors:** 


### OBSTETRIC MEDICINE.

1. A NEW SYMPTOM OF RUPTURE OF THE UTERUS.—Dr. M'Clintock related some cases of rupture of the uterus and of the vagina, to illustrate the obscurity which may occasionally surround the diagnosis of this accident when occurring during parturition. He particularly drew attention to a symptom observed in one of the cases, which he considered might hereafter be found of value as a diagnostic of laceration of the uterus or vagina. This symptom was *an emphysematous state of the integuments covering the hypogastrium.* Its existence was detected by the stethoscope while searching for the fœtal heart. Examined for in this manner, the crepitation was loud and distinct, but to the hand it was not so obvious, except when firm pressure was made in the proper situation, then the crepitus was evident, and was recognized by Dr. G. Montgomery, and by several pupils who happened to be present at the case. The other symptoms present were of so ambiguous a kind, that the possibility of rupture having taken place seemed doubtful. There was no prostration, no recession of the presenting part, and no cessation of the pains; the woman was able to be up and to walk about. Having ascertained that the emphysema had not extended downward from the chest or neck, and that it was confined to the suprapubic and iliac regions, Dr. M'Clintock inferred that the air had obtained an entrance through some rent in the genital passages. Acting upon this conclusion, the woman was delivered by craniotomy, (the fœtal heart had not been heard for two hours previously.) She sank rapidly after delivery, and died in a few hours. The left broad ligament was found emphysematous, and a tear existed in the left side of the uterus at the junction of the body and cervix. At some distance from this the peritoneum was found rent also, and a considerable quantity of blood had been effused into the abdominal cavity.—*Dublin Quarterly Journal; Edinburgh Medical Journal*, February, 1858.

2. Puerperal Convulsions. By *Dr. Pirrie.* (Read before the Belfast Medical Society.)—I have long been dissatisfied with the *routine practice*, and its results, in cases of puerperal convulsions. By routine practice I need scarcely say that I refer to bleeding, which in its various forms has been recommended most urgently by almost all of even our modern authorities in obstetrics.

Thus, we everywhere meet with expressions such as the following: "Bleeding is our great reliance." "The lancet is our sheet-anchor; and blood may be taken to a very large extent," (Ramsbotham.) "If there be a case in which the bold and daring use of the lancet is demanded, it is the case of puerperal convulsions," (Meigs.) "The first thing to be done is to take away blood from the arm or temporal artery largely and in a full stream," (Churchill.) "In the first rank both as prophylactic and curative are to be placed sanguine emissions," (Cazeaux.) But that such was the universal practice, I need cite no authority, as I believe all of us have been in the habit of thus treating our cases of puerperal convulsions, as I was myself till some time ago, when I happened to have a case of convulsions occurring during the progress of Bright's disease of the kidney in a male, and a case of puerperal convulsions in a pregnant female before labor, under my care about the same time. I was then most forcibly struck with the similarity of the signs and symptoms in the two cases: in both there was œdema of the face and upper extremities, accompanied by albuminuria, and the paroxysms of convulsion in both were absolutely identical. I treated them both on the same principles, of which bleeding formed no part, as being virtually different stages of the same disease, and had the satisfaction of seeing the case of puerperal convulsions recovering without a trace of remaining disease; and the case of Bright's disease, although looked upon at one time as actually moribund, so far recovered as to be able to leave the hospital in decidedly an improved state.

In the book just published by Herr Braun, Professor of the Imperial Midwifery Clinique at Vienna, (the chapter of which on convulsions has been translated by Dr. Duncan of Edinburgh,) he formally protests against the practice of bleeding in cases of puerperal convulsions, and says that "general depletion of blood has very seldom any valuable effect on the symptoms, and generally produces irreparable injury." The same author also affirms that *every* case of *true* puerperal convulsion, or eclampsia vera puerperarum, is dependent on *uræmia*, that is, on the blood being contaminated by urea, and perhaps also by other excrementitious products which should have been excreted by the kidneys, had they been properly discharging their function.

This important fact in pathology, like almost all other discoveries in medicine, appears to have been arrived at by degrees. Thus, as early as the time of Hamilton it was observed that women who during pregnancy had œdema of the face or upper extremities, were very liable to have convulsions at the time of labor. Following this up, Simpson of Edinburgh, in 1843, found that the secases were associated with the presence of albumen in the urine; and in 1848 he demonstrated the presence of disease of the kidney at the post-mortem of a case of puerperal convulsions; and he also observed the presence of albumen in the urine of a child affected with convulsions, born of a mother similarly affected. But I believe it has been reserved for Professor Braun first publicly to declare that every case of *true eclampsia* arises from uræmia, and thereupon to modify the treatment of the disease.

As the term eclampsia is generally applied to an acute affection of the motor function of the nervous system, characterized by tonic and clonic spasms, and insensibility, and as several different pathological states produce these phenomena, and yet have nothing further in common, and have not the same influence upon the life of the mother or offspring,—it becomes of the utmost importance to make a true and particular diagnosis of these different forms of convulsions.

Agreeing with Professor Braun, I would restrict the term true puerperal convulsions (which may occur during pregnancy any time after five months, during parturition or childbed, or even later) to those cases dependent on *uræmia.* The term uræmia is still retained, although it is admitted that the convulsions do not originate, as was formerly supposed, from the blood being poisoned by urea, as filtered urine has been injected into the veins of animals without producing convulsions.

After a series of carefully performed experiments, Frerichs was led to the conclusion that the phenomena of uræmic intoxication are not produced by urea or any other ingredient of the urine, but that they commonly arise from this circumstance, that the urea, accumulated in the blood, is transformed into carbonate of ammonia, under the influence of some peculiar ferment. For the production of uræmic phenomena, it is therefore necessary to have in the blood urea in quantity, and also some ferment, by means of which the urea may be changed into carbonate of ammonia. If the ferment be wanting, then the blood may for a long time be impregnated with urea without any convulsions appearing; in this way is accounted for the fact that in the bodies of persons dead of Bright's disease, the blood may be found saturated with urea, without any uræmic phenomena having been observed during life. Simpson has most ingeniously suggested that it may be to this principle of action that the beneficial effects of chloroform inhalations are due in uræmic convulsions, as chloroform produces a temporary diabetes mellitus, and it has been demonstrated that sugar in minute quantities, added to the urine, prevents for a time the natural decomposition of urea into carbonate of ammonia.

Among other convulsive affections which may occur during pregnancy or labor,

I would include hysteric, epileptic, and apoplectic convulsions, and convulsions arising from other impurities (bile, carbonic acid) retained in the blood, or from poisons admitted into the circulation, (as lead, mercury, arsenic,) and anæmia. The possibility of any of these forms of convulsions (differing essentially in their prognosis and treatment) occurring during labor, amply shows that too much importance cannot be attached to the *differential diagnosis* in all cases of puerperal convulsions.

The true pathology of eclampsia (till 1848, when Simpson first showed the co-existence of granular degeneration of the kidneys) was unknown; the usual remark being, that post-mortem examination afforded but little information, there generally being no deviation whatever from the healthy state of the brain. But since attention has been directed to the renal origin of puerperal eclampsia, I believe that in almost every fatal case that has been minutely examined, one or other of the three stages of Bright's disease has been constantly found, corresponding to the description given by Dr. Bright, and recently and more minutely by Frerichs, and by Dr. Johnson in his valuable work on Diseases of the Kidney.

It is not necessary that I should take up time describing these pathological changes, which must be familiar to all, from the many specimens of diseased kidney exhibited in connection with Bright's disease. I would rather direct attention to the signs of the existence of this disease during life: I mean those changes observed in the urine and the œdematous condition of the face and upper extremities, which, as I have already remarked, has long been observed as a precursor of convulsions; but dropsical swelling is not necessarily connected with albuminuria, just as, on the other hand, there occurs during pregnancy a dropsy in which the urine is found quite normal.

Hyperæmia of one or both kidneys, caused by congestion of venous blood, is the primary stage of acute Bright's disease. This is soon followed by fibrinous exudation into the Malpighian corpuscles, the albumen only of this exudation at first passing off with the urine, while the fibrinous matter, coagulating in the tubuli of the cortical substance, and remaining in them for a longer or shorter period, is at length propelled from them, along with the exfoliated epithelium, in the form of cylindrical casts of the tubes. Hence we have, during life, first the urine charged with albumen, which may be discovered with the usual tests; and afterwards, the casts of the tubes, which can only be discovered by microscopic examination.

In testing for albumen we should be careful to use moderately diluted nitric acid, and not the strong fuming acid, as the latter decomposes and redissolves the albumen. And we should also bear in mind that it is only in urine having an *acid* reaction that albumen is precipitated in quantity by boiling; for in alkaline urine the *ammonia*, which is always present, retains the albumen in solution. I have known the presence of albumen to have escaped observation from the neglect of these simple precautions.

Different explanations have been given as to the cause of the renal hyperæmia, the most plausible ascribing it to congestion produced by pressure of the gravid uterus on one or both renal veins. Of cases of eclampsia, above 80 per cent. (96 per cent., Collins,) occurred in *primiparæ*, in whom, on account of the greater resistance of the abdominal walls, a more powerful counter-pressure is produced on the kidneys. Eclampsia, too, is frequently met with where there is increased pressure from plural pregnancy, dropsy of the amnion, deformed pelvis, or excessive obliquity of the uterus. Something also is, no doubt, due to the altered state of the blood during pregnancy.

*Treatment.*—Should albuminuria be diagnosed during pregnancy, something may be done by way of *prophylaxis*, by the administration of such medicines as will prevent the decomposition of the urea, or rather will neutralize the carbonate of ammonia. According to Frerichs, benzoic acid is the most efficient remedy, and the free use of drinks acidulated with lemon-juice or tartaric acid. If the secretion of urine be very scanty, the occasional use of purgatives will be useful as preventing local congestion. But as long as pregnancy continues, we can only expect the mitigation of the albuminuria, not its cure, the cause still being present; and should symptoms arise indicating immediate danger to life, or producing serious functional derangement of the heart, brain, or lungs, the propriety of inducing artificial premature labor should then be seriously entertained.

When convulsions have actually occurred, we have to consider the medical and obstetrical treatment.

The medical treatment will be the same whether the convulsions occur during pregnancy, labor, or childbed,—the first object being to diminish as much as possible the reflex excitability of the nervous system, and to weaken the paroxysms so as to gain time. As the best means of obtaining these results, all who have used chloroform inhalation speak of it in the highest terms, its success surpassing all expectations. It should be administered immediately upon indications of an impending paroxysm presenting themselves. The inhalation should be kept up till the premonitory symptoms of the paroxysm disappear—which is generally in the course of a minute or so—but, should it not be possible to cut short the paroxysm, the chloroform inhalations should be suspended during the paroxysm and supervening coma, for obvious reasons.

The beneficial effects of chloroform may be ascribed to its sedative effect on the nervous system, or to its peculiar action on the blood, as suggested by Simpson, in arresting the further decomposition of the urea.

In the interval of the paroxysms the direct medical treatment of the uræmia should be proceeded with by administering five to ten-grain doses of benzoic acid, and cold acidulated drinks. If the bowels have been constipated, and the paroxysms severe, a bolus with five to ten grains of calomel, with a drop or two of croton oil, followed by turpentine injections, will generally be found useful. To moderate the secondary congestions of the head, which come on after the paroxysms, the local application of ice or the cold douche will be found to have a more powerful and lasting influence than the use of leeches.

Sponging the skin with tepid vinegar is stated to be very useful in producing diaphoresis. As a rule, general depletion should be avoided, as it very seldom produces any valuable effect on the symptoms, and generally produces irreparable injury. If, however, a cautious selection of single cases be made, one moderate bleeding in strong, full-blooded women may not only not be injurious, but may much facilitate the action of other remedies.

Revulsive measures, as sinapisms to the calves of the legs and soles of the feet, and blisters to the nape of the neck, are generally recommended, but I do not think that any very marked benefit can be expected from them.

But whatever benefit may be derived from any of these measures, we must still hold the prompt and careful evacuation of the uterus to be a most essential and important point in the treatment of puerperal convulsions. How this is to be accomplished will, of course, depend upon whether uterine action has commenced or not, or upon the stage which the labor has reached. Should the paroxysms continue after the evacuation of the uterus, and the administration of benzoic acid and cold acidulated drinks, opium given in doses of one to three grains, or one-eighth to one-half grain doses of morphia, with opiate injections, generally acts most beneficially.—*Dublin Quarterly Journal of Medical Science*, February, 1858.

3. STATISTICS OF OPERATIVE MIDWIFERY.—This is a report by the great Leipsic veteran, *Dr. Meissner*, on the results of his thirty-five years' midwifery practice, as far as operative midwifery is concerned. He observes, that a review of private midwifery practice must be taken from quite a different point of view to that of a public institution; for while in the latter the bulk of the cases are examples of natural labor, the private practitioner is only consulted, as a general rule, when the case has become pathological. [These observations are, of course, only applicable to the mischievous continental practice of leaving cases in private practice to the almost exclusive care of midwives, the physician only being called in when the case has assumed a serious aspect, and, of course, frequently too late.] Moreover, in the Lying-in Hospital, the physician treats his cases in a suitable locality, with all he wants at hand; while in private, the practitioner is called miles away to neglected cases, having insufficient appliances, and is forced to treat his cases under the most disadvantageous circumstances.

Dr. Meissner's observations relate to 3811 women, who, he says, gave birth to 3980 children, as 136 were twin, and 2 triplet births. But there is here obviously some error in the numbers, as these do not correspond with each other. Of the 136 twin-births, in 54 there were 2 boys, in 35, 2 girls, and in 47, children of both sexes. Of the triplets, in one case there were 2 boys and 1 girl, and in the other, 3 girls. These 3811 labors called for operative interference in 3025 instances, dynamic aid being required in 924 of these to effect change of position. There were 1863 forceps operations (!), this instrument being required in 5 other instances for the removal of separated fungoid or polypous tumors. Turning was performed 351 times, extraction 247 times, perforation 32 times, the induction of premature labor 20 times, the Cæsarean operation 6 times, *accouchement forcé* 55 times, polypus operations 6 times, placenta separation or deliveries 447 times; 2086 boys and 1684 girls were born at full time, while there were 265 premature births or abortions, and 24 moles and polypi; 399 children were born dead, and of those living, 36 died within fourteen days. Twenty-five mothers died either before or during labor, or within sixteen days after it.

*Turning.*—Although this, when performed at the proper time, is usually an easy operation, executed within a few minutes, yet of the author's 351 cases, in only 222 did it prove completely successful for both mother and child. In 104 cases the child was born dead, in 2 cases the mother died from the delivery, and in 2, after this had taken place. This issue is chiefly to be attributed to the ignorance of midwives in the detection of cross presentations, and the delay that elapsed before aid was obtained. Dr. Meissner prefers turning by one foot, unless there is some indication for hastening the delivery. This rule is of especial importance when multiple births are expected, so as to avoid getting hold of two feet of separate children. In one of the author's cases of triplets all the children presented cross-wise, and six lower extremities were felt. All the children were safely delivered by operating upon one foot at a time. The one-foot delivery also gives the child the best chance of being born alive. *Cephalic* version is preferred by some practitioners, as giving the child the best chance; but it can only be tried when hastening the delivery is not an object. The author resorted to it successfully in 6 cases. In 4 of the 351 cases turning was performed by *external manipulation*, and in 3 by *spontaneous* version, the buttocks being forced by the presenting shoulder into the pelvis, and all the children being born dead.

*Forceps Operations.*—Of 1863 cases, in 1750 the head was the presenting part; and in 113 the forceps were used for its delivery in other presentations. Of the 1754 children delivered, 99 were born dead, *i.e.* nearly 12·19. This, in an operation which, carefully performed, can hardly be considered dangerous to life, is a high proportion; but the child had, in fact, not unfrequently died during pregnancy, and was in advanced putrefaction on delivery. In 10 instances the child was hydrocephalic or dropsical; in 3 the mother was already dead; there were 5 cases of spina bifida; 7 deaths took place from prolapsed funis, and 3 from the arm and head being tightly wedged in together. In 4 instances the mother had suffered from repeated convulsive paroxysms. In other cases the application of the forceps had been repeatedly attempted by preceding practitioners, or the passage was narrowed by the presence of tumors. Dr. Meissner remarks, by-the-way, that the separation of the epidermis is not always a certain sign of the death and putrefaction of the child, as it may be produced by an acrid condition of the *liq. amnii.*

*Extraction.*—This was performed in 247 cases, and became necessary when, in breech knee, or foot presentation, the child's life was threatened by cessation of pain, faulty position, or prolapsus of funis. Only 145 of the children were born living; but then, 13 had died through pressure on the funis, before the author's arrival; 18 were in a state of putrefaction, 10 were already born except the head, 8 were immature, and in 5 others there was hydrocephalus, or other form of dropsy. In 42 instances extraction had to be performed on account of cessation of pain after turning.

*Perforation.*—In his thirty-six years' practice, this operation has only been performed by the author thirty-two times, and he had attended 3299 labors before he had his first case. He has always followed the maxim laid down by the chief German practitioners, of never proceeding to the operation until assured of the child's death; and it has several times happened to him to see living children born in cases which have been left for days together to the powers of nature, and which in previous labors had been delivered by perforation.

*Premature Labor.*—So much has the performance of the operation for premature labor limited that of perforation, that while to the year 1842 he had had to resort to perforation in twenty-eight instances, he has during the subsequent fourteen years only performed it four times. He has never induced premature labor prior to the thirty-sixth week, and has never found this too late, the bones continuing thus long sufficiently soft and yielding to accommodate themselves to the narrowed pelvis. When the reckoning is uncertain, he performs the operation thirty-six weeks after the last menstrual period.

*Forced Delivery* (*Accouchement Forcé*).—By this term the author understands the whole series of operations (as artificial dilatation of the os uteri, bursting the membranes, turning, extraction, or removal of placenta,) which may be required for delivery when the further continuance of pregnancy is dangerous to mother and child. It is especially called for in certain cases of eclampsia, placenta prævia, and obstinate vomiting. He enumerates under this head two instances of opening the adherent os uteri by means of the knife, and eight cases of its forcible dilatation. This last procedure was resorted to because, after the labor had continued three or four days, in place of dilatation of the os uteri, general debility and delirium set in. The author has resorted to it fifty-five times during thirty-six years. In thirty-three of these cases both mother and children did well, although, as the dilatation was usually undertaken for placenta prævia, most of the latter were born some weeks too soon. As the majority of cases (31) were examples of placenta prævia, in which hemorrhage had continued long before the patients were seen by the author, it is not surprising that ten of the mothers died; but another statement of the author, that even when he arrived at his patients he plugged the vagina, and awaited pains before proceeding to deliver, is somewhat extraordinary.

*Cæsarean Operation.*—Of the six examples of this that have fallen to the author's lot, five were peformed on mothers being already dead, the children being saved in none. In the case of operation upon the living subject, both mother and child lived.

*Placenta Removals.*—The number of these (447) must seem very large; but it is to be remarked that perhaps not one-tenth of these cases were examples of abnormal adhesion requiring separation, the placenta being frequently detained from other causes, preventing the success of the ordinary manipulations, as "stricture of the uterus," spasmodic contraction of the uterus from too early interference, etc.

*General Results.*—Of the mothers, 41 were lost; 25 during, and 16 after delivery. Of the former 25, 11 were already lifeless when seen; and of the 14 others, 1 died from rupture of the omentum with internal hemorrhage, 10 from placenta prævia necessitating forced delivery, 2 from nervous shock after favorable labor, and 1 from hemorrhage. Of the 16 mothers who died after delivery, 1 died from cancer of the stomach, 1 from pneumonia, 3 after repeated attacks of eclampsia, 1 from putrescence of the uterus, 1 from typhus following the birth of a putrid, premature child, 4 from puerperal fever following operative procedures, 1 from "paralysis of the lungs," 3 from the consequences of loss of blood, and 1 after several hours' operative attempts by a country practitioner.

Of the *children* 399 were born dead, as already stated, under the various operations. Besides these, 36 died within the first fourteen days after birth; viz., 4 from debility from too early birth, 6 from atelectasis pulmonum, 2 from trismus, 1 from fissure of the cranium after a forceps operation, 1 from chronic hydrocephalus, and 2 from want of breast-milk.

After remarking upon the remarkable sequences met with in practice of unusual pathological occurrences, and of the operations required for their relief, the author observes, that, as a general rule, forceps operations are found to be most frequent in cold, changeable weather, which induces rheumatic affections of the uterus, not only rendering the dilatation of the os very painful, but delaying its accomplishment for days. This condition may be often prevented by clothing warmly the lower part of the person; and when it is present it should be treated by Dover's powder. The standing too much over the fire, also, may, by over-heating the abdomen, lead to a plethoric condition of the anterior wall of the uterus, which may not be without its influence in inducing morbid adhesion of the placenta. Such an occurrence is best prevented by abstaining from this practice, and bathing the abdomen with cold water. When adhesion has taken place repeatedly at the same place, in consequence of an indurated condition of a portion of the uterine wall, we should, after the termination of the puerperal condition, endeavor to induce absorption by mild mercurial or iodine frictions, tepid baths, together with hemlock and mallow injections. If these do not succeed, the baths at Krankenheil, near Tolz, in Bavaria, which have been found so useful in fibroid and hypertrophy of the uterus, should be tried. —*Monatschrift für Geburts-Künde,* band ix., pp. 19–72.—[*Med. Times and Gazette,* May 29, 1858.]

4. INFLUENCE OF PREGNANCY AND DELIVERY UPON INSANITY. By *M. Marcé.* —M. Marcé thus concludes an interesting paper illustrated by cases. 1. We cannot protest too strongly against the practice of those physicians who advise or allow pregnancy in insane women, for it results from the facts mentioned in this paper that, in the great majority of cases, pregnancy and delivery, so far from exerting a favorable influence on insanity, seem, on the contrary, to hasten on the progress of the disease toward dementia. If in certain exceptionable cases (2 in 16) pregnancy has suspended the progress of the disease, the improvement has been only temporary, and the insanity has reappeared after delivery. 2. In some few cases (4 in 16) remarkable especially for the predominance of erotic symptoms, pregnancy has exerted a beneficial influence on the cure. 3. When insanity becomes developed during pregnancy, it very often remains incurable, even after delivery, or is cured so long after that no influence can be attributed to the latter in the termination of the nervous affection. 4. Sometimes, however, (3 in 10 cases,) the disease disappears after delivery, and these cases must be regarded as sympathetic. 5. Delivery in the insane is often remarkable for the slight amount, or even complete absence, of pain.—*Annales Medico-Psychol.,* tome iii., p. 359.—[*Med. Times and Gazette,* April 10, 1858.]

